# Formation of the junctions between lymph follicles in the Peyer's patches even before postweaning activation

**DOI:** 10.1038/s41598-024-65984-4

**Published:** 2024-07-09

**Authors:** Anri Teshigahara, Yuri Banba, Hiromi Yoshida, Mitsuji Kaji, Zhou Zhou, Nao Koyama, Yoshifumi Sakai, Niel A. Karrow, Kouetsu Ogasawara, Ryota Hirakawa, Jahidul Islam, Mutsumi Furukawa, Tomonori Nochi

**Affiliations:** 1 International Education and Research Center for Food and Agricultural Immunology, Graduate School of Agricultural Science, Tohoku University, Miyagi, 980-8572 Japan; 2 Laboratory of Animal Functional Morphology, Graduate School of Agricultural Science, Tohoku University, Miyagi, 980-8572 Japan; 3 Institute of Development, Aging and Cancer, Tohoku University, Miyagi, 980-8575 Japan; 4 Department of Animal Biosciences, University of Guelph, Ontario, N1G 2W1 Canada; 5 Laboratory of Animal Mucosal Immunology, Graduate School of Agricultural Science, Tohoku University, Miyagi, 980-8572 Japan; 6 Division of Mucosal Vaccines, International Vaccine Design Center, The Institute of Medical Science, The University of Tokyo, Tokyo, 108-8639 Japan; 7 Center for Professional Development, Institute for Excellence in Higher Education, Tohoku University, Miyagi, 980-8576 Japan

**Keywords:** Peyer’s patches, Lymphoid follicles, Germinal centers, 3D analysis, Amira, Mucosal immunology, Lymphoid tissues, 3-D reconstruction

## Abstract

Peyer’s patches (PPs), which contain an abundance of B and T cells, play a key role in inducing pivotal immune responses in the intestinal tract. PPs are defined as aggregated lymph follicles, which consist of multiple lymph follicles (LFs) that may interact with each other in a synergistic manner. LFs are thought to be spherical in shape; however, the characteristics of their structure are not fully understood. To elucidate changes in the structure of PPs as individuals grow, we generated serial 2D sections from entire PPs harvested from mice at 2, 4, and 10 weeks of age and performed a 3D analysis using a software, Amira. Although the number of LFs in PPs was not changed throughout the experiment, the volume and surface area of LFs increased significantly, indicating that LFs in PPs develop continuously by recruiting immune cells, even after weaning. In response to the dramatic changes in the intestinal environment after weaning, the development of germinal centers (GCs) in LFs was observed at 4 and 10 weeks (but not 2 weeks) of age. In addition, GCs gradually began to form away from the center of LFs and close to the muscle layer where export lymphatic vessels develop. Importantly, each LF was joined to the adjacent LF; this feature was observed even in preweaning nonactivated PPs. These results suggest that PPs may have a unique organization and structure that enhance immune functions, allowing cells in LFs to have free access to adjacent LFs and egress smoothly from PPs to the periphery upon stimulation after weaning.

## Introduction

Peyer’s patches (PPs), which are secondary lymphoid tissues of the small intestine, consist of follicular and interfollicular regions, each respectively containing primarily B and T cells^1,2^. PPs act as hubs for the induction of T cell-dependent antigen-specific immune responses. Under the direction of antigen-specific T cells, B cells differentiate into IgA class-switched plasmablasts that ultimately become IgA plasma cells, which are abundantly present throughout the intestinal lamina propria where IgA is secreted and transported into the intestinal lumen^3–6^. IgA is secreted from two types of plasma cells that have different B-cell origins. PP-derived IgA plasma cells originated from conventional B cells (B2 cells) are present in the intestinal lamina propria where nonconventional B cells (B1 cells)-derived IgA plasma cells originated from the plural and peritoneal cavities are also localized^7,8^. These B1 cells are considered part of the innate immune system and are capable of secreting “natural antibodies”, including IgA, as antigen-naïve B cells^9^. Because T cell-dependent immune responses are predominantly induced by B2 cells in PPs^7^, the idea of targeting vaccine antigens to PPs has been widely accepted as a potential means to counter enteric pathogens by inducing antigen-specific IgA production through immunizations such as oral vaccination. With recent advances in gene technology analysis, the diversity of B-cell receptors (BCRs) and T-cell receptors (TCRs) in PPs can be examined by sequencing those genes that B and T cells possess^10^. In addition, flow cytometry can be used to count absolute numbers of B and T cells in PPs^11^. However, the histological features that allow efficient monitoring of antigen-specific immune responses, such as antigen recognition, and antigen-specific interactions between T and B cells in PPs, and subsequent B-cell proliferation and differentiation have not yet been fully elucidated.

PPs are aggregated lymph follicles as opposed to isolated lymph follicles (ILFs) that also develop in the intestinal tract. The characteristics of PPs and ILFs are different with respect to organogenesis. Specifically, PPs develop prenatally in cooperation with T and B cells^12,13^, whereas ILFs are formed postnatally, primarily by B cells^14,15^. In PPs, T cells essentially reside in the interfollicular regions and associate with the follicular regions, which are preferentially composed of B cells^1^. The molecular mechanism by which the interfollicular and follicular regions develop is explained by the interaction of chemokines and their receptors, CCL19/CCL21 and CCR7, and CXCL13 and CXCR5, respectively^16–19^. Although their structures (e.g., the volume of the follicular region) are thought to be ingeniously configured to promote efficient T and B cell functions, few studies have examined the relationship between the function and morphology of PPs. In addition, most previous studies of PP organogenesis have focused on development during the early embryonic stage^20,21^. Therefore, further research is needed on the postnatal effects of milk, microbes, nutrients, and other factors on PP formation. In particular, differences in the functional morphology of PPs observed before and after weaning should be investigated in detail in relation to changes in the intestinal environment.

One of the major challenges of histological analysis of circular tissue is the difficulty in establishing the position of the tissue sections in the overall tissue used for analysis. Information obtained from the 2D analysis is very different when analyzing the center and margins of the tissue structure. The multiple lymph follicles that form PPs are thought to be circular in shape; however, most studies analyzing PP function using histological observations have been conducted based on information obtained from randomly generated 2D sections. Nevertheless, it should be noted that PPs have been reconstituted based on images obtained from 2D serial sections in a limited number of previous studies. Specifically, using a series of programs (Image J, Reconstruct, 3D Studio Max), the volume of lymph follicles (LF) and germinal centers (GF) of PPs reconstructed as 3D images from 2D serial sections were measured^22^. Another image processing package (Fuji) based on the images created by Image J has allowed for a more accurate understanding of the distribution and frequency of B, T, and dendritic cells in the 3D reconstructed PP images^23^. In addition to 3D construction using 2D images, transparency techniques have recently made it possible to visualize the structure of whole tissues, even allowing the structure of circular tissues to be examined, and such techniques been used to analyze the structure and function of PPs^24,25^. A limitation of this study, however, is that the reactivity of antibodies used for immunohistochemistry (IHC) is much higher for 2D tissue sections than for whole 3D tissues, even with such sophisticated techniques. Given the continues improvements in software for combining 2D images for performing 3D analysis, more detailed 3D images that resemble intact tissue will be created by using the latest software for 3D reconstruction in combination with advanced skills for creating multiple serial sections.

Therefore, in this study, numerous serial 2D tissue sections of PPs harvested from 2-, 4-, and 10-week-old mice were prepared. Subsequent histological images were used to reconstruct 3D images using a software Amira to examine the changes in the functional morphology of PPs, especially before and after weaning.

## Results

### Establishing a 3D analysis strategy to understand the functional morphogenesis of PPs

**Figure 1:**
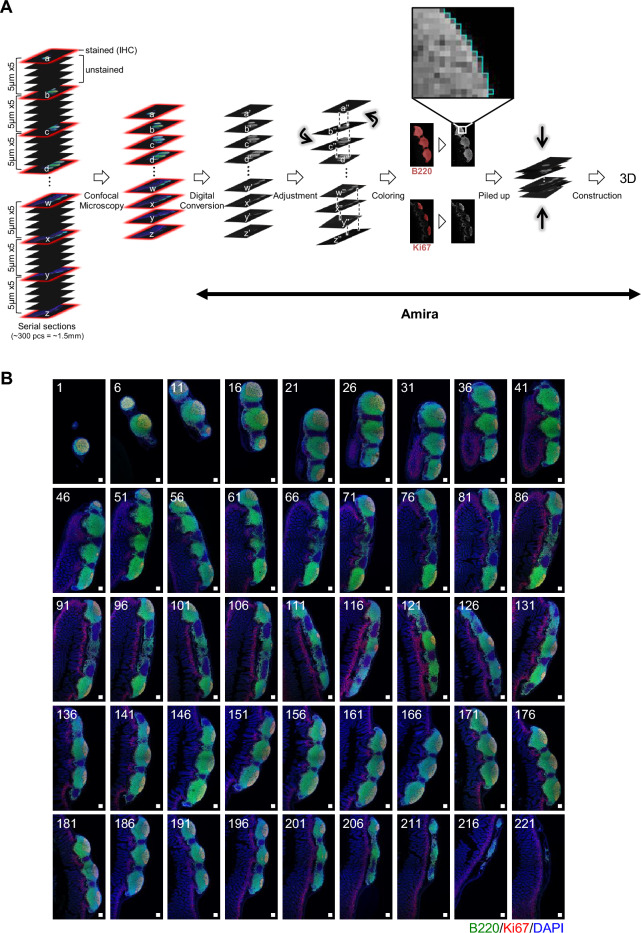
Procedure of the 3D analysis used to study the functional morphology of PPs. (**A**) Serial tissue sections of PPs were harvested and prepared from the jejunum; one in five sections was selected for IHC using anti-B220 and anti-Ki67 antibodies, and images were obtained using a confocal microscope (TCS SP8). Images were digitally converted using Amira software by adjusting torsion and skipping every four of five sections to create an accurate 3D image. (**B**) Representative images were processed for 3D analysis. Then, 45 images obtained from at 5-section intervals were used for 3D analysis to create a 3D image of PPs at 10 weeks of age. Scale bars = 100 µm.

To overcome the limitations of a 2D analysis, which usually analyzes limited regions within an entire tissue, a procedure was developed using Amira to reconstruct the 3D structure of PPs based on multiple entire images obtained from 2D serial tissue sections. Specifically, from the more than 200 serial sections prepared from each PP, 1 in every 5 was selected for this analysis. Images of specific regions of each section were obtained from confocal microscopy using lenses with either 10 × or 20 × magnification, which were further combined using a tiling module to create the entire image of each 2D tissue section. It should be noted that Amira can precisely connect the junction of two associated upside-down images, even if some images between the two have been omitted from the analysis (Fig. 1A). In a representative study to investigate PPs at 10 weeks of age, 45 tissue sections were selected from 221 serial sections to create 3D images (Fig. 1B and Supplementary Fig. 1). The sections were stained with anti-B220 and anti-Ki67 antibodies to detect B cells and proliferating cells, respectively, and to visualize germinal centers (GCs) that include Ki67^+^ proliferating cells, which are densely located within lymph follicles (LFs) that are composed mostly of B220^+^ B cells in PPs. Amira provided accurate positional information of the structures within PPs, such as GCs, as 3D coordinates. Therefore, this technique was used to perform a further comparative study to investigate the morphological uniqueness of PPs as individuals grow.

### Changes in the functional morphogenesis of PPs before and after weaning

**Figure 2:**
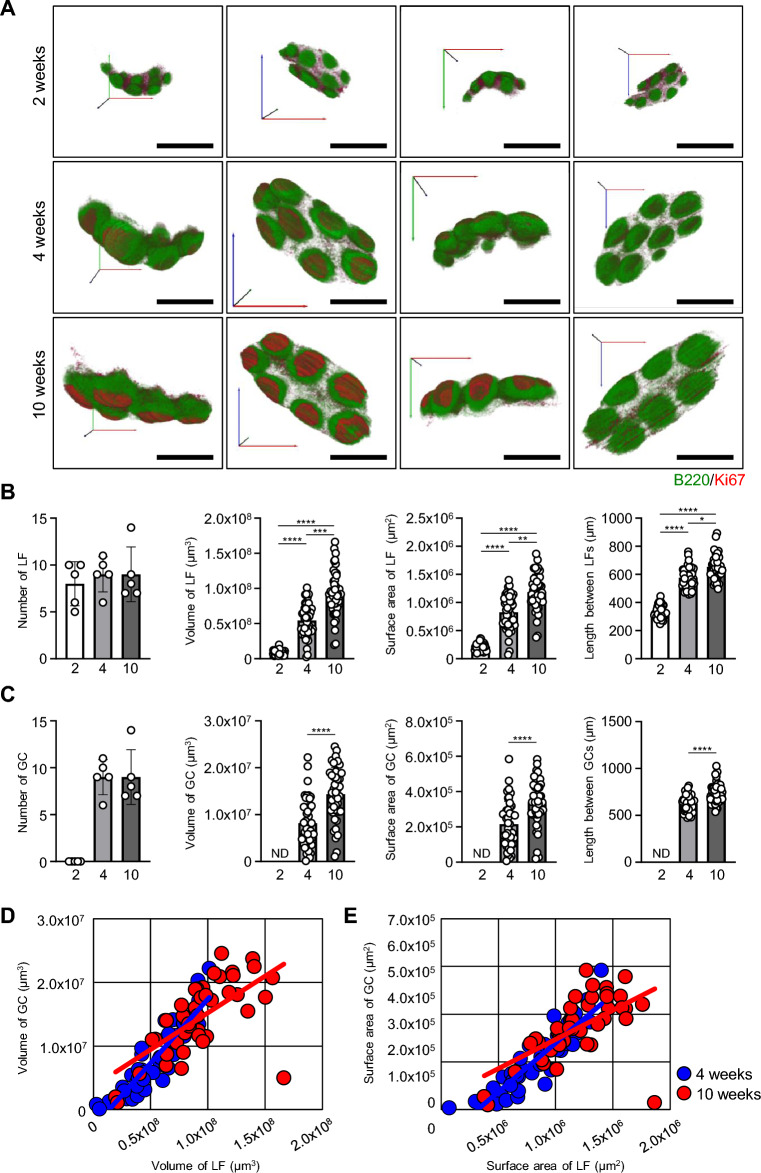
Enlargement of PPs after birth and formation of GCs after weaning at 4 and 10 weeks of age. (**A**) Representative 3D images of PPs at 2, 4, and 10 weeks of age. A single PP is shown from four different angles. Green: LFs containing B220^+^ B cells. Red: GCs including Ki67^+^ proliferating cells. In this figure, the obtained images were overlaid with each registered color without manual marking, constructing a 3D image as fine dots. (**B**) The number, volume and area of LFs, and length between LFs were investigated at 2, 4, and 10 weeks of age. (**C**) The number, volume and area of GCs, and length between GCs were investigated at 2, 4, and 10 weeks of age. (**D**) Positive correlations between LF and GC volumes at 4 and 10 weeks of age. (**E**) Positive correlations between LF and GC surface area at 4 and 10 weeks of age. Scale bars = 1 mm. Kruskal–Wallis test (**B**) or t-test (**C**) was conducted to make comparisons among the three groups (2, 4, and 10 weeks of age) or between two groups (4 and 10 weeks of age), respectively. Blue and red circles indicate results at 4 and 10 weeks of age, respectively. *p < 0.05, **p < 0.01, ***p < 0.001, ****p < 0.0001.

In this study, PPs were harvested from a total of 15 mice at 2, 4, and 10 weeks of age. Five mice were used at each stage and one representative PP was harvested from the same region of the small intestine. Serial sections were prepared from each PP and subjected to IHC. Consistent with the knowledge that PP organogenesis initiates prenatally, the structure of LFs could be visualized, even at 2 weeks of age (Fig. 2A and Supplementary Video 1). No obvious GCs containing Ki67^+^ cells were observed at 2 weeks of age. Ki67^+^ cells were abundant in the interfollicular region but were not obvious at 4 and 10 weeks of age (Fig. 2A). The formation of LFs in PPs was not induced because the number did not change throughout the developmental process (Fig. 2B). The analysis using 3D coordinates in Amira showed that the volume and surface area of LFs, and center-to-center length between related LFs increased significantly with growth (Fig. 2B). Because all LFs in PPs formed GCs after weaning, the number of GCs in LFs did not differ between 4 and 10 weeks of age (Fig. 2C). Nevertheless, the volume and surface area of GCs, and center-to-center length between GCs were much greater at 10 weeks than at 4 weeks of age (Fig. 2C). There was a positive correlation between the volume and surface area of LF and GC within each LF at 4 and 10 weeks of age (Fig. 2D,E). These results indicate that PPs begin to function by forming GCs in response to post-weaning stimuli, and the PP structure further develops significantly for at least 6 weeks.

### Visualization of GCs by detection of B220^+^Ki67^+^ cells in PPs

**Figure 3:**
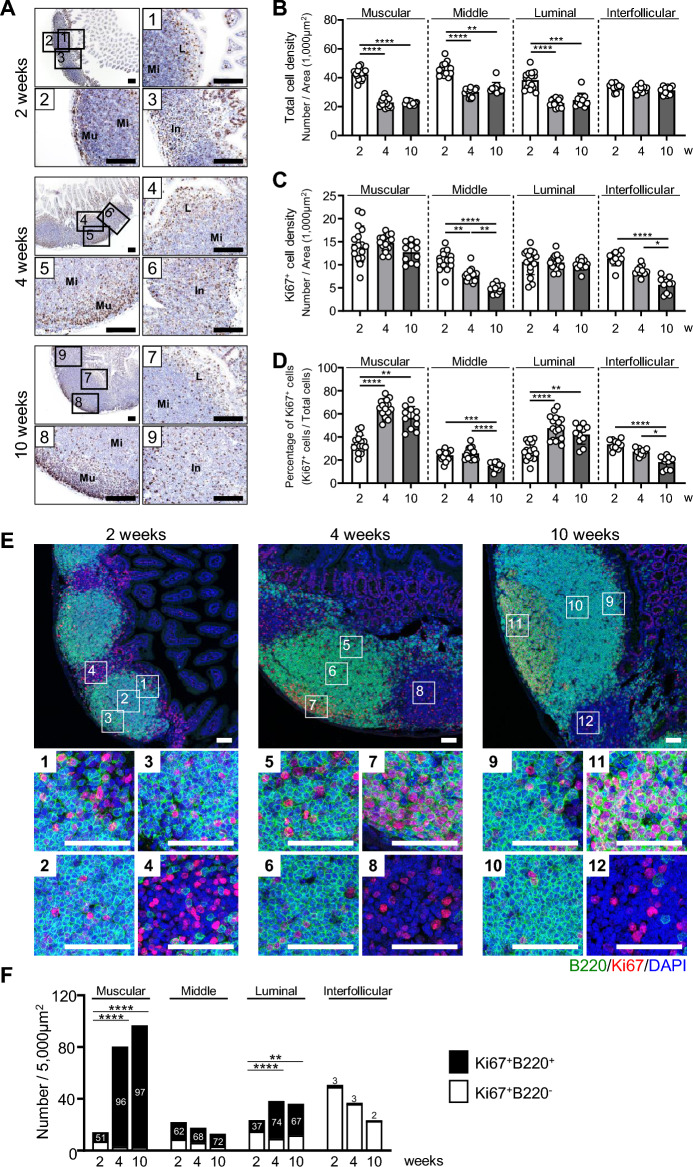
Distribution of Ki67^+^ cells and B220^+^ in PPs at 2, 4, and 10 weeks of age. (**A**) The intestinal lumen side was defined as luminal (L); the muscular layer side as muscular (Mu); the intermediate between the two as middle (Mi); and the region between LFs as interfollicular (In). The number of total cells (**B**), the number of Ki67^+^ cells (**C**), and the percentage of Ki67^+^ cells to total cells (**D**) in each region were shown. (**E**) Tissue sections of PPs were stained with anti-B220 and anti-Ki67 to determine the distribution of proliferating and non-proliferating B cells in the luminal (1, 5, 9), middle (2, 6, 10), muscular (3, 7, 11), and interfollicular (4, 8, 12) regions. (**F**) The numbers of Ki67^+^B220^+^ and Ki67^+^B220^−^ cells in each area at 2, 4, and 10 weeks of age were shown. Scale bars = 100 µm (**A**) and 50 µm (**E**). Kruskal–Wallis test was conducted to make comparisons among the three groups (2, 4, and 10 weeks of age). *p < 0.05, **p < 0.01, ***p < 0.001, ****p < 0.0001.

After confirming the localization of Ki67^+^ cells microscopically, a quantitative histological analysis was performed to calculate the number of Ki67^+^ cells and to quantify the percentage of total cells within certain areas of 2D sections prepared from the center of the tissue at 2, 4, and 10 weeks of age. A general IHC using anti-Ki67 antibodies showed that GCs with densely accumulated Ki67^+^ cells were only found in PPs at 4 and 10 weeks, especially in areas close to the muscle layer (Fig. 3A). Some Ki67^+^ cells were also scattered throughout the PPs at all time points analyzed, regardless of the presence or absence of GCs (Fig. 3A). Therefore, to better understand changes in the number of Ki67^+^ cells and the percentage of total cells without defining them as clustered or scattered, the tissue sections were divided into four compartments (i.e., luminal, middle, muscular and interfollicular regions) to perform a quantitative analysis for each area within PPs. At 2 weeks of age, the density of total cells was significantly greater in the luminal, middle, and muscular regions compared with the density at 4 and 10 weeks of age (Fig. 3B). Interestingly, the number of Ki67^+^ cells and the percentage of total cells in the interfollicular region reached a maximum at 2 weeks of age and then significantly decreased thereafter (Fig. 3C,D). Consistent with the fact that proliferating B cells are involved in the formation of GCs after weaning, more than 95% of Ki67^+^ cells were B220^+^ B cells in GCs present in the muscular side of PPs at 4 and 10 weeks (Fig. 3E,F, and Supplementary Fig. 2). Ki67^+^ cells observed in the interfollicular region were mostly B220^−^ non-B cells (Fig. 3E,F, and Supplementary Fig. 2). Based on these results, we defined the region containing densely assembled B220^+^Ki67^+^ cells as GCs in our subsequent 3D analysis to investigate the change in the functional morphogenesis of PPs.

### Adhesion of adjacent LFs in PPs

**Figure 4:**
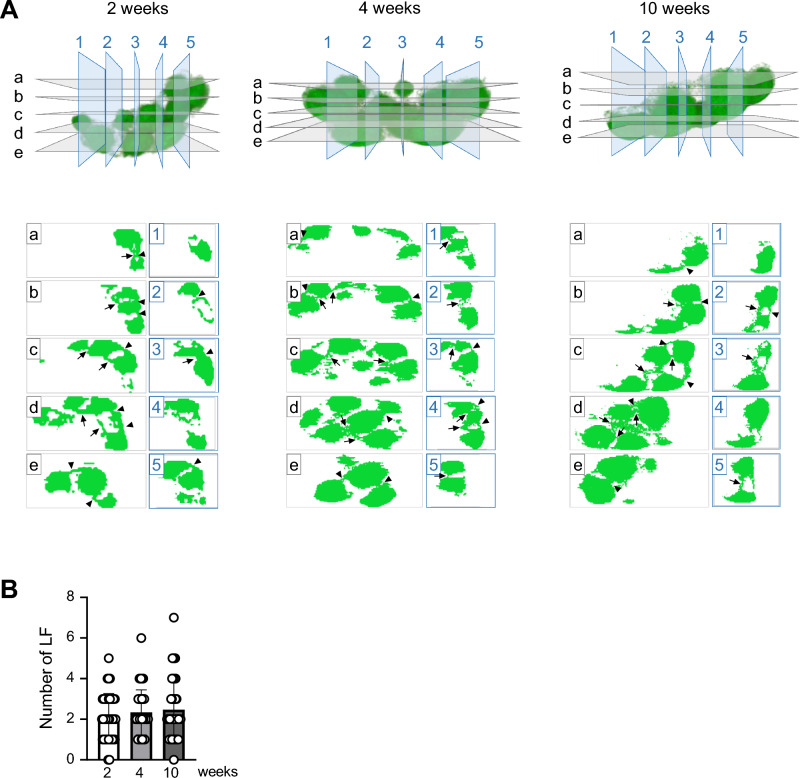
The formation of junctions between LFs in PPs at any timepoint in the analysis. (**A**) The horizontal plane shows a sectional view from above the displayed 3D image, and the vertical plane shows a sectional view from the right. (**B**) The number of adjacent LFs to which each LF in a PP connects. Arrow: junction between LFs on the luminal side. Arrowhead: junction between LFs on the muscle layer side.

Amira allowed us to scan the constructed 3D digital images from different angles to create various 2D virtual images that helped us to characterize the morphological uniqueness of PPs. In longitudinal and transverse scanned 2D images showed that LFs containing B220^+^ B cells adhere to adjacent LFs through attachment points in PPs (Fig. 4A, Supplementary Fig. 3, and Supplementary Video 2). Importantly, the development of extracellular matrix composed of collagen fibers was barely found at the attachment points. These results suggest that the presence of such physical barriers is unlikely to interfere in the migration of B cells from one LF to an adjacent LF (Supplemantary Fig. 4). In addition, there was little evidence of a relationship between the formation of attachment points and the enlargement of LFs in PPs during development because attachment points were constantly observed at 2, 4, and 10 weeks of age. Given that most LFs associate with adjacent LFs in PPs, the number of attachment points mostly depends on the number of adjacent LFs (Fig. 4A). Furthermore, the number did not differ with age (Fig. 4B). These results suggest that, from a very early stage, immune cells (mainly B cells) accumulating in LFs may have direct access to adjacent LFs via attachment points rather than through the circulatory system via blood or lymph vessels.

### Changes in the center of gravity of GCs in the LFs of PPs during the developmental process

**Figure 5:**
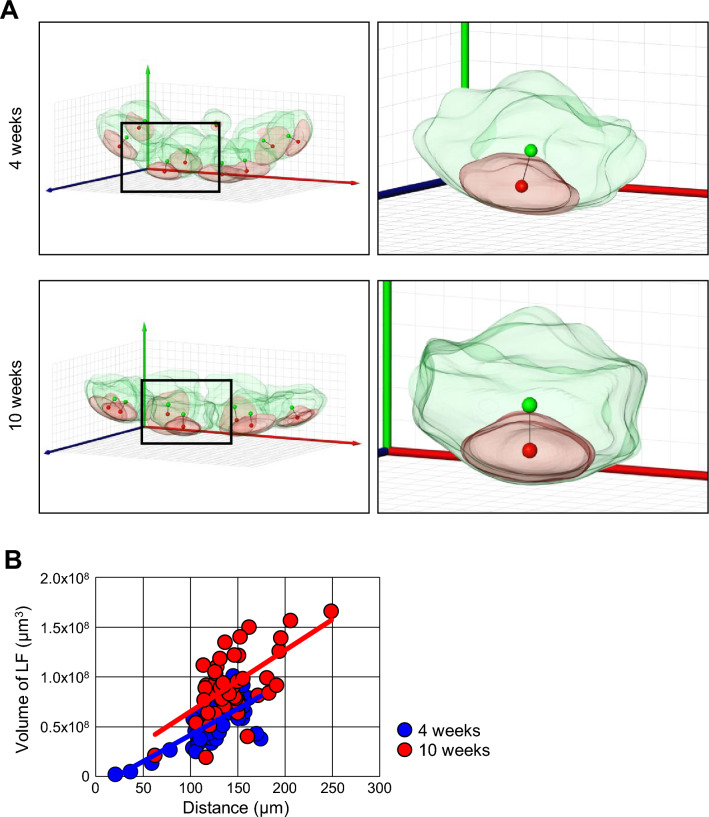
Misalignment of the center of gravity of LFs and GCs. (**A**) The centers of gravity of LFs and GCs at 4 and 10 weeks of age were shown as green dots and red dots, respectively. (**B**) A correlation between the volume of LFs and the distance between the center of gravity of LFs and GCs was shown. Blue and red circles indicate results at 4 and 10 weeks of age, respectively.

**Figure 6:**
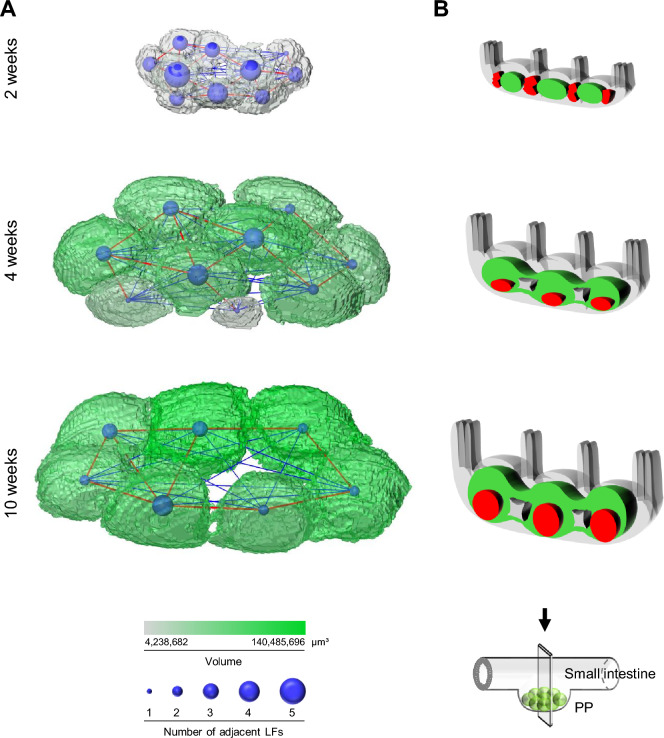
Morphology of PPs visualized in this study as shown by the 3D analysis. (**A**) The volume of LFs is indicated by a green color gradient. The number of junctions in each LF was indicated by the size of the ball displayed at the center point. (**B**) GCs, shown in red, which form in LFs, shown in green, develop significantly near the muscle layer after weaning.

The advantage of this study using Amira is that the myriads of coordinate axes from the surface of the structure can be obtained in digital files. Therefore, to understand the structural changes in PPs with GC formation during the developmental process after weaning, the centers of gravity points of GCs and LFs were determined individually as 3D coordinates using the reconstructed 3D images of PPs created at 4 and 10 weeks of age. Representative PP images, with the muscular layer side placed on the bottom and the intestinal lumen side placed on the top, showed that the center of gravity of each GC and the corresponding LF did not coincide at either 4 or 10 weeks of age, indicating that GCs do not form at the center of LFs in PPs (Fig. 5A and Supplementary Video 3). Importantly, a positive correlation existed between the volume of LFs and the distance from the center of gravity of the GCs to that of LFs (Fig. 5B). These results suggest that GCs may form away from the center of LFs as PPs develop after weaning. To summarize our findings using Amira, a network analysis study was conducted to visualize our discoveries. In the representative PPs at 2, 4, and 10 weeks of age, least one to five attachment points were present in each follicle, regardless of age (Fig. 6A and Supplementary Video 4). No relationship was found between the number of attachment points possessed by each follicle and their localization within PPs. There was a persistent change in the structure of PPs after weaning, presumably because GCs more often formed in the muscular side of PPs (Fig. 6B). These results suggest that this persistent change in morphology may be important for understanding the functional maturation of PPs.

## Discussion

One of the key findings obtained in this study was the adhesion of adjacent LFs in PPs; generally, individual LFs were thought to be independent, as they are surrounded by connective tissue and interfollicular regions^26,27^. The number of attachment points that connect adjacent LFs did not change throughout the experimental period. These results suggest that the features of LFs in PPs were not induced, for example, by the formation of the gut microbiota after weaning. One possible hypothesis to explain why LFs adhere to adjacent follicles is that B cells present in PPs can have free access to migrate from one LF to adjacent LFs. If this is the case, it is also important to consider the need for B cells to freely change their position in PPs. As the most useful feature of Amira is its ability to calculate the volume of LFs from 3D images reconstructed from numerous 2D images obtained from serial tissue sections^28^, we attempted to predict the number of cells in LFs to better understand the structural features of PPs. It should be noted that the volume of B cells was assumed to be 523.6 µm^3^ as the typical diameter of a B cell is 10 µm. Given that the volume of LFs at 10 weeks of age was 78,280,668.47 μm^3^, as shown in Fig. 2, the maximum number of cells present in the LFs was estimated to be 149,505 if intercellular spaces are not considered. Considering the number of PPs, and the number of LFs in each PP, which was approximately 9 at 10 weeks of age, as shown in Fig. 2, there are more than 10^7^ cells in the LFs present in all PPs.

Another important consideration is the antigen recognition diversity of B cells in PPs. It should be noted that there are more than 10^11^ theoretical patterns of B cell receptor (BCR) repertoires with different complementarity-determining regions in murine B cells. Of these, B cells with 10^8^ patterns, excluding the self-antigen specific BCR repertoires, are believed to be present, especially within secondary lymphoid tissues, including PPs^29,30^. Therefore, it is possible to hypothesize that B cells may freely reposition themselves, even after migrating into LFs through attachment points to access adjacent LFs, not only to enhance the efficacy of antigen recognition in each follicle but also for the efficient antigen-specific interaction with T cells in PPs.

PPs are generally divided into four regions (i.e., follicular-associated epithelium, FAE; subepithelial dome region, SED; follicular region; and interfollicular region) based on the individual characteristics of the functional morphology and the localization of different types of cell populations^17,31^. The FAE is comprised of microfold (M) cells along with regular epithelial cells that selectively sample luminal antigens, such as bacteria^6,32–34^. Dendritic cells process these antigens in the SED to present antigen-derived peptides to naïve T cells that are abundant in the interfollicular region^35,36^. Naïve B cells primarily accumulate in the follicular region, where immune responses can occur after antigen-specific interactions with T cells^6,37^. In this study, Ki67^+^ cells were predominantly clustered in the muscular region of PPs forming GCs. However, numerous Ki67^+^ cells were also observed scattered throughout PPs. Of these cells, the relative number of Ki67^+^ cells in the SED increased after weaning and these cells were mostly B cells. These results suggest that this activation of B cells in the SED is presumably facilitated mostly by the recognition of microorganisms via M cells after weaning and is further stimulated by interaction with antigen-specific T cells. Given that GCs are only observed after weaning in PPs, it is reasonable to consider that such an increase in the presence of Ki67^+^ B cells in the SED may therefore be involved in the formation of GCs. Another interesting observation in this study showing 3D reconstituted PPs was that GCs developed in PPs on the muscular side rather than at the center of LFs. Activated B cells differentiate into plasmablasts during the formation of GCs^38^. In addition, an abundance of lymphatic vessels develops in the connective tissue on the muscle side^39^, facilitating the migration of plasmablasts from PPs toward the intestinal lamina propria where the cells further differentiate into plasma cells producing antibodies, mostly IgA^40,41^. Further studies are needed to address the molecular mechanism by which GCs develop unevenly within LFs; however, it can be hypothesized that B cells may change their position within PPs depending on the various stages of activation as they proliferate.

Through the comparison of pre- and post-weaning, this study found that the total number of cells present in certain areas of the follicular region decreased after weaning, but not in the interfollicular region. Interestingly, this feature was observed in all three areas of the follicular region (i.e., luminal, middle, and muscular areas), regardless of the presence or absence of GCs. B cells circulating in blood vessels accumulate in the follicular region of PPs through high endothelial venules (HEVs)^18,42,43^. The expression of CXCL13 by HEVs, which are mesenchymal organizer cells, and by follicular dendritic cells plays a key role, as it is the necessary chemokine for recruiting B cells into the LFs of PPs^17,44,45^. Therefore, together with the corresponding receptor CXCR5 expressed by B cells, CXCL13 and CXCR5 are primarily responsible for cell tropism into PPs^17^. To the best of our knowledge, no studies have shown a difference in cell density of PPs between preweaning and postweaning. Nevertheless, based on our findings shown in Fig. 3, we propose that the formation of LFs in PPs occurs in two sequential stages: the recruitment of B cells into PPs via CXCL13/CXCR5-dependent chemotaxis, which occurs before weaning^45,46^, and the formation of the extracellular space surrounding the B cells and the consequent decrease in B-cell density that occurs after weaning. Given that the follicular region of PPs develops conduits that allow the flow of absorbed luminal fluid to be directed^47^, it may be hypothesized that the development of conduits that reduce cell density may also be promoted after weaning in PPs. Furthermore, our 3D analysis showed that the number of Ki67^+^ non-B cells was greater in certain areas of the interfollicular region before weaning (at 2 weeks of age) than after weaning. Although the function of proliferating non-B cells in the interfollicular region needs to be elucidated by further study using, for example, single-cell analysis, such cells may contribute to the independent formation of multiple LFs in PPs, especially during development, thereby enabling an efficient immune response after weaning.

The acquired immune system of newborns is quiescent during nursing but begins to develop after weaning^48^. Breast milk contains several molecules, some of which may suppress the early stages of immune development, especially in the gastrointestinal tract, as they are transferred from the mother to the offspring^49,50^. In addition, microorganisms are known to play important roles in the functional maturation of PPs, as germ-free mice form immature PPs^51,52^. Given the dramatic differences in the pre- and postweaning microbiota in the presence or absence of milk supply^53^, it will be of significant importance to identify specific factors in the milk and gut microbiota that promote immunosuppression within PPs before weaning and further maturation of PPs after weaning, respectively. In particular, as certain microorganisms such as *Alcaligenes* spp. have been identified as commensal bacteria in PPs^54^, it may also be important to investigate changes in the microbial environment within PPs, especially before and after weaning. Although age-related changes in the functional form of PPs were beyond the scope of this study, it should be noted that impairment of PP function due to a decrease in functional M cells in aged mice has also been reported^55^. Thus, it is possible that the features of PPs identified in this study, such as adhesion to adjacent LFs and formation of GCs at specific sites, are lost in aged mice. As mentioned above, conventional 2D analysis based on classical histochemistry is limited to providing sufficient information for a deeper understanding of tissue structure. Therefore, there is a growing need for 3D analysis using analytical tools, such as Amira, not only to accurately understand the structure of PPs during nurturing, development, and aging but also to support the development of stage-specific therapeutics to promote and maintain the functional formation of PPs.

## Materials and methods

### Animals and samples

BALB/c mice (2, 4, and 10 weeks old) were maintained at the animal facility of the Graduate School of Agricultural Science, Tohoku University. All animal experiments were designed in accordance with protocols approved by the Tohoku University Experimental Animal Committee. Experiments were performed according to ARRIVE and institutional guideline.

### Histological analyses

PPs in the middle part of the small intestine were harvested from BALB/c mice euthanized at 2, 4, and 10 weeks of age. The tissues were fixed overnight in 4% (w/v) paraformaldehyde and embedded in paraffin. Serial tissue Sects. (5 µm) were prepared using a microtome and subjected to IHC. Specifically, the sections were first treated with Target Retrieval Solution (Dako) at 98 °C for 40 min for antigen retrieval and incubated with 0.5% (w/v) blocking reagent (Perkin Elmer) for 30 min at room temperature. To detect B cells and proliferating cells in PPs, the sections were stained with either purified rat anti-mouse B220 (1:100, RA3-6B2, BD Bioscience) or purified rabbit anti-Ki67 (1:200, polycolonal, Abcam) antibodies overnight at 4 °C. After washing, sections stained with rabbit anti-Ki67 were treated with Histofine Simple Stain MAX PO (R) (undiluted, Nichirei Biosciences) and signals were developed with 3,3′-diaminobenzidine tetrahydrochloride. Some sections stained with anti-mouse CD45R followed by Histofine Simple Stain Mouse MAX PO (Rat) (undiluted, Nichirei Biosciences) were further stained using Van Gieson solution (Nippon RiKa). Some sections stained with both rat anti-mouse CD45R and rabbit anti-Ki67 were treated with Alexa Fluor488-labeled donkey anti-rat IgG (1:100, Jackson ImmunoResearch) and Alexa Fluor647-labeled donkey anti-rabbit IgG (1:100, Jackson ImmunoResearch) antibodies for 1 h at room temperature. Finally, the sections were counterstained with either hematoxylin (Wako), or DAPI (DOJINDO). Tissue images were obtained using two microscopes, BX-63-DP72 (Olympus) and TCS SP8 (Leica Biosystems), and entire images were created by tiling. In some experiments, Ki67^+^ and Ki67^+^B220^+^ cells were counted using ImageJ software (NIH). Specifically, the total number of cells present in the luminal, middle, muscular, and interfollicular regions in specific areas (1000 μm^2^) was counted.

### 3D analysis using Amira

The acquired images were loaded into Amira software (Thermo Fisher Scientific) for 3D visualization and analysis to superimpose the images. Specifically, one out of every five serial sections were used for 3D reconstruction. LFs are defined as the accumulation of B220^+^ B cells and GCs are defined as the accumulation of Ki67^+^ cells in the muscle region. LFs and GCs were gated manually on Amira and distinct images were created (Supplementary Fig. 1). The images with a vertical relationship were automatically connected and superimposed by simulating the presence of four unused tissue sections between the captured images. The volume and surface area of the LFs and GCs were calculated from the 3D images created by Amira.

### Statistical analysis

Statistical analyses were conducted using Prism 7 software (GraphPad) to perform *t*-tests for the results in Fig. 2C and multiple comparison tests in Figs. 2B, 3B–D, F and 4B). All statistical details are provided in the figure legends, with *p* values shown in the figures.

### Supplementary Information

Supplementary Figures.

Supplementary Video 1.

Supplementary Video 2.

Supplementary Video 3.

Supplementary Video 4.

## Data Availability

All data involved in this study are available from the corresponding author upon request.
