# Supplementary material for: Formation of the junctions between lymph follicles in the Peyer's patches even before postweaning activation
Source: Sci Rep. 2024 Jul 9;14:15783. doi: 10.1038/s41598-024-65984-4 (PMC11233632; doi:10.1038/s41598-024-65984-4)
Supplement: Supplementary file 1 — Supplementary Figures. [file 41598_2024_65984_MOESM1_ESM.pdf]

A

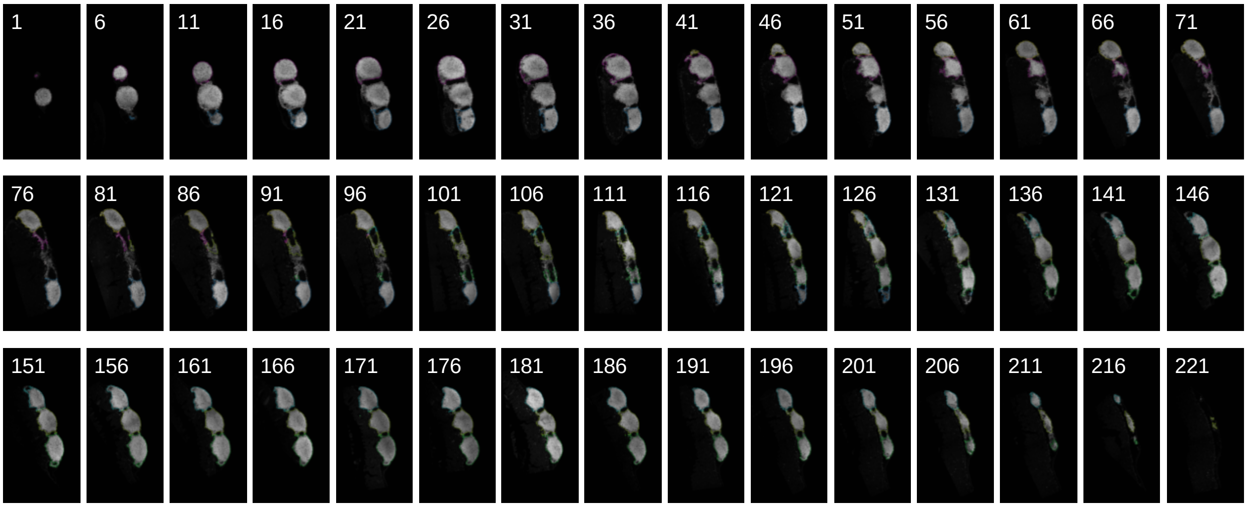

B

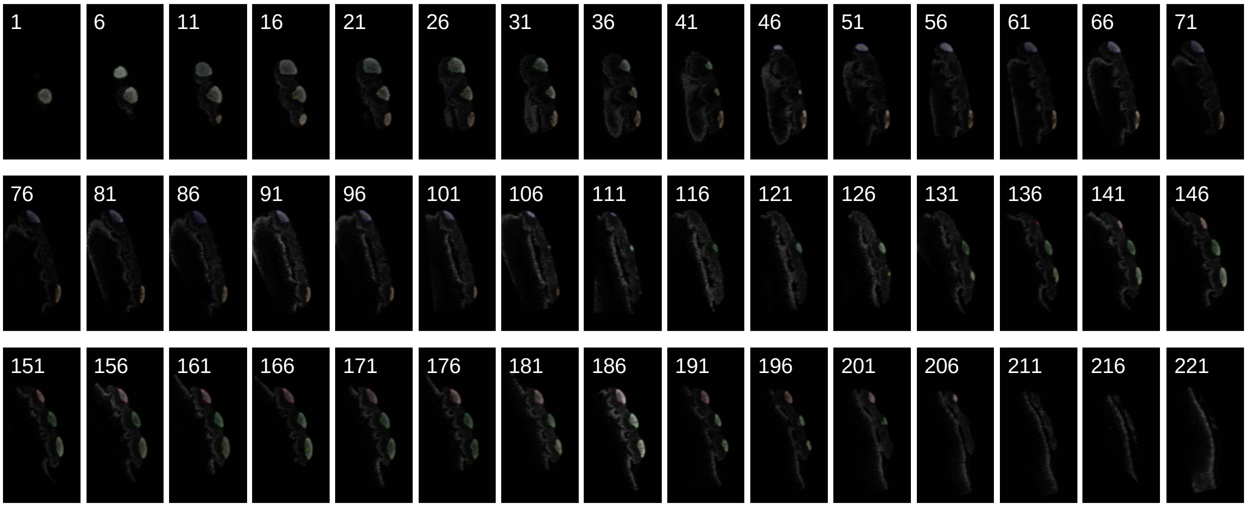

Supplementary Figure 1. Compartmentalization of LFs and GCs using Amira. (**A**) Areas containing B220$^+$ B cells in individual images were marked as LFs. (**B**) Areas of Ki67$^+$ cell accumulation in the region proximal to the muscle layer in individual images were marked as GCs. Scale bars = 100 µm.

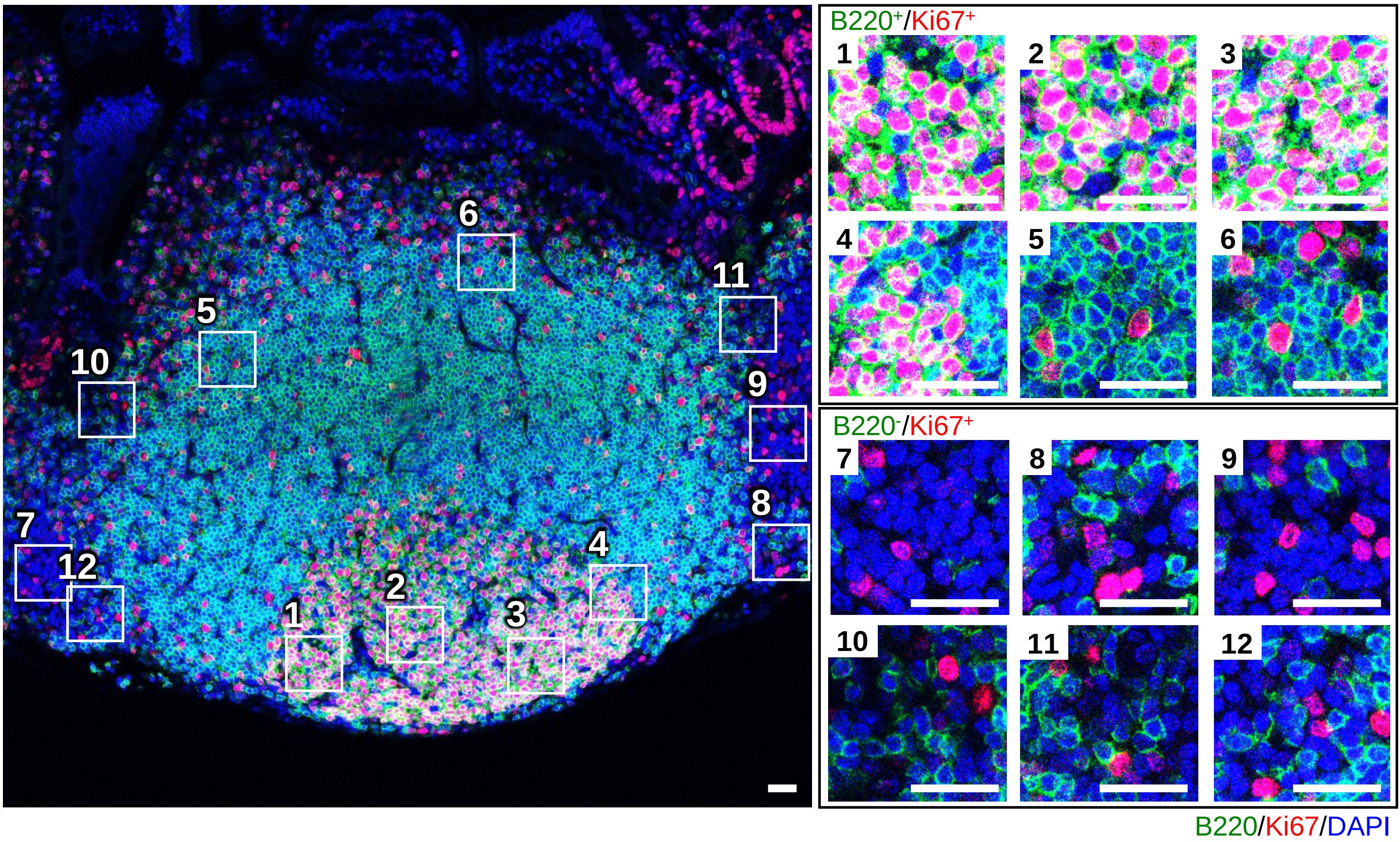

Supplementary Figure 2. Presence of B220⁺Ki67⁺ cells in GCs at 10 weeks of age. Ki67⁺ cells in GCs were mostly B220⁺ B cells, whereas Ki67⁺ cells in the interfollicular region were mostly B220⁻ non-B cells. Scale bars = 25 µm.

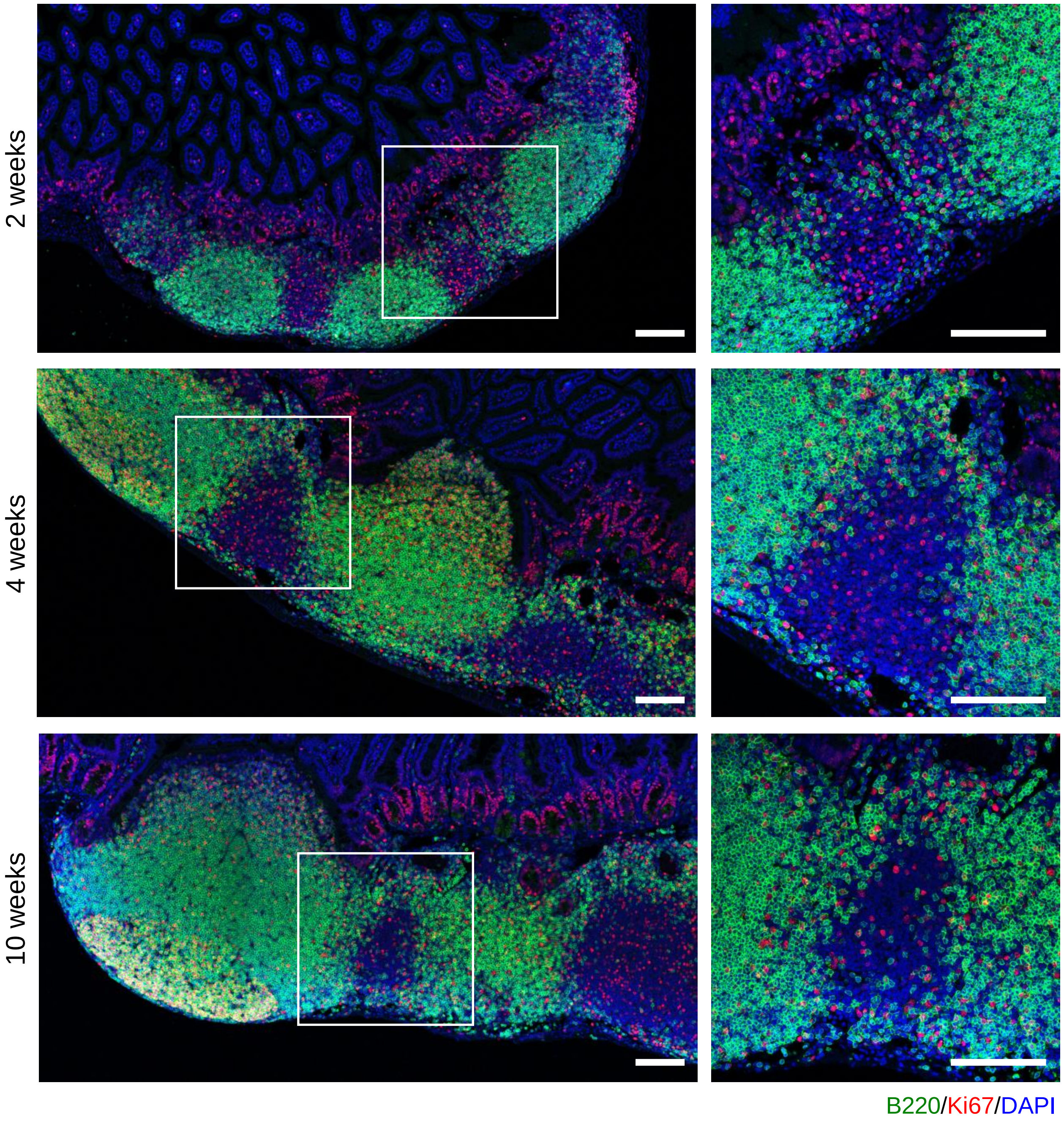

Supplementary Figure 3. The formation of junctions between LFs in PPs. B220^{+} B cells were found between LFs at any timepoint in the analysis. Scale bars = 100 µm.

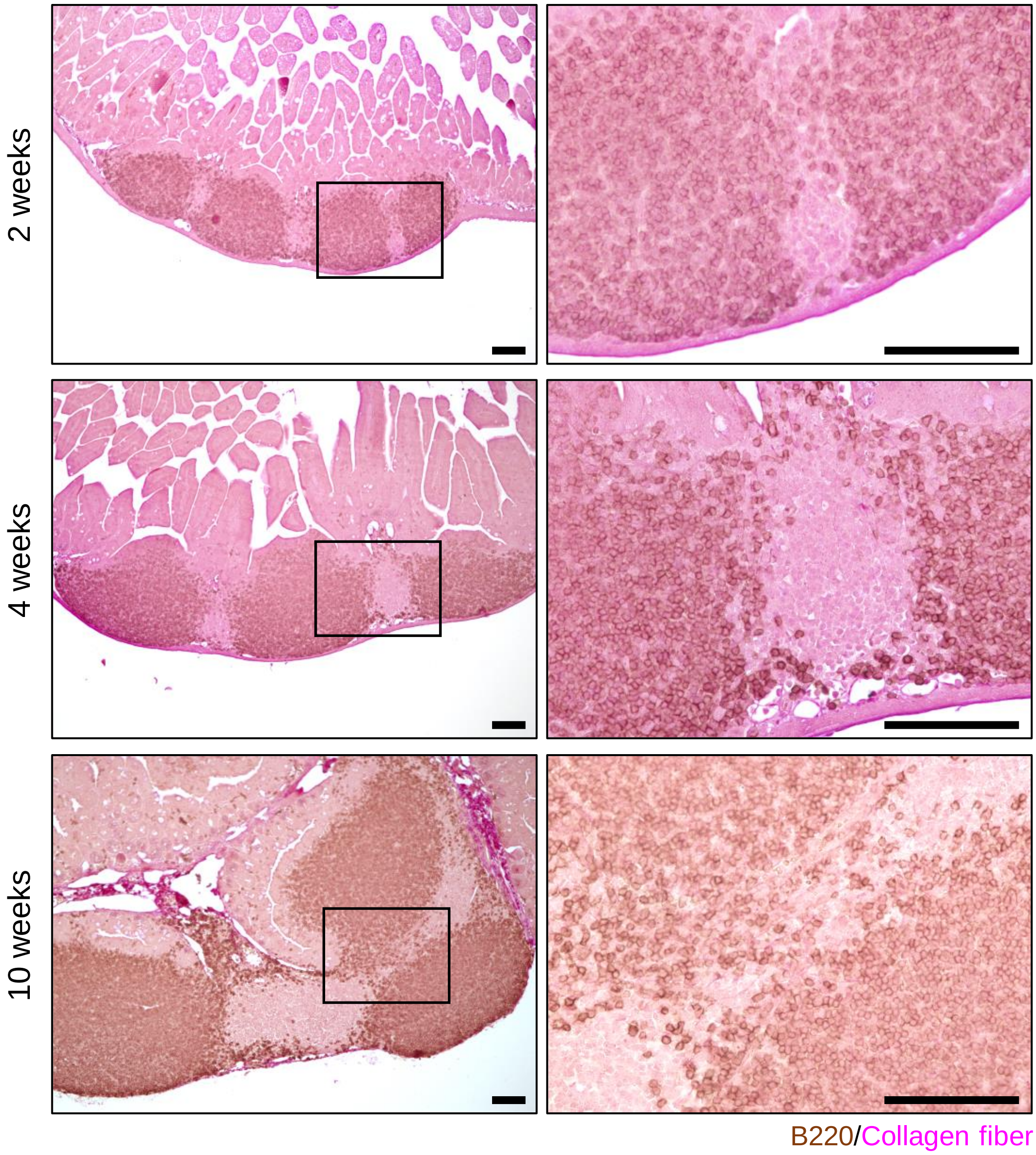

Supplementary Figure 4. Distribution of extracellular matrixes composed of collagen fibers and B220^{+} B cells in PPs. Red and brown indicate collagen fiber and B220^{+} B cells, respectively. Scale bars = 100 µm.
