# Supplementary material for: Formation of the junctions between lymph follicles in the Peyer's patches even before postweaning activation
Source: Sci Rep. 2024 Jul 9;14:15783. doi: 10.1038/s41598-024-65984-4 (PMC11233632; doi:10.1038/s41598-024-65984-4)
Supplement: Supplementary file 2 — Supplementary Video 1. [file 41598_2024_65984_MOESM2_ESM.pptx]

## Slide 1
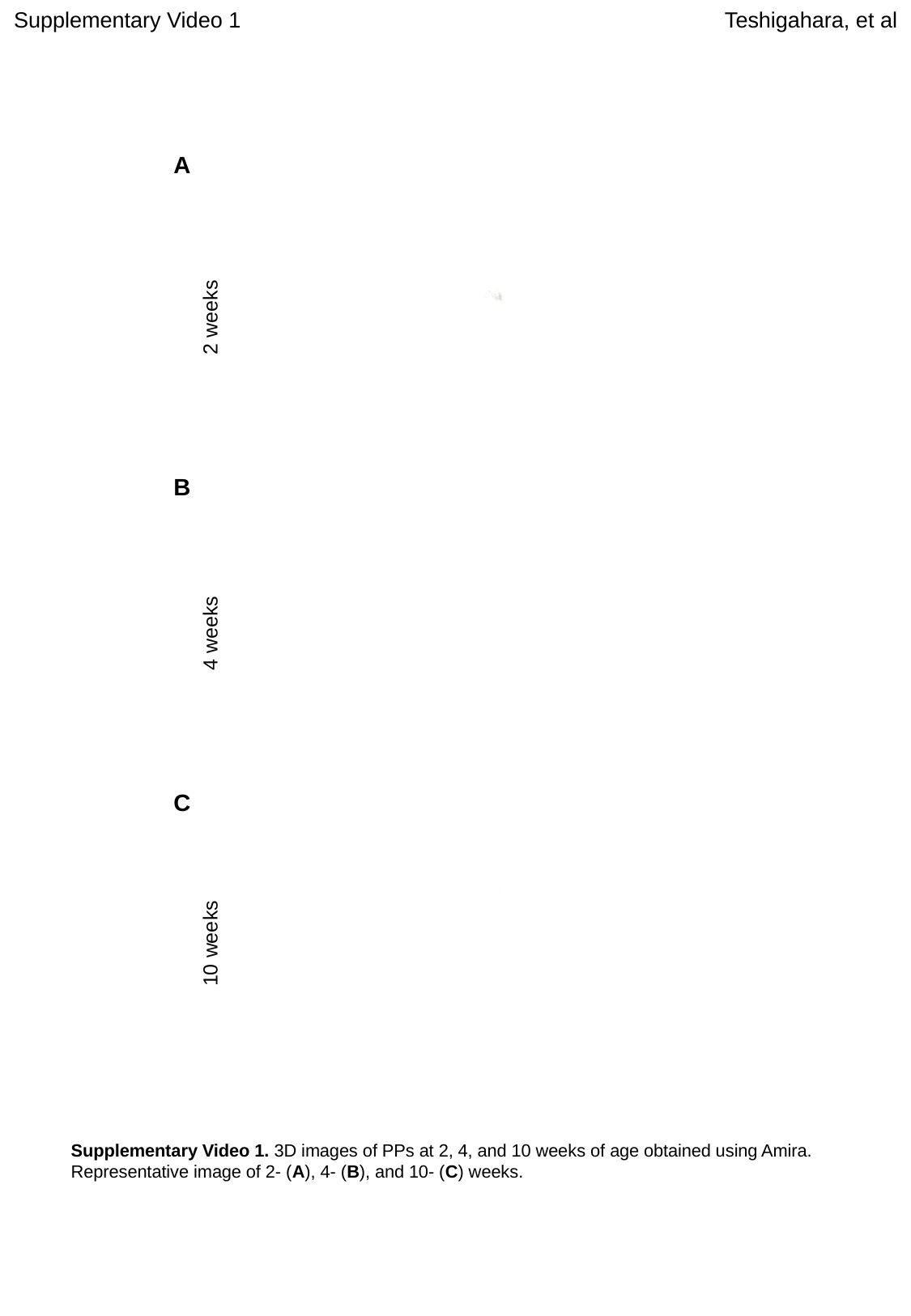

Supplementary Video 1
Teshigahara, et al
A
2 weeks
B
4 weeks
C
10 weeks
Supplementary Video 1. 3D images of PPs at 2, 4, and 10 weeks of age obtained using Amira. Representative image of 2- (A), 4- (B), and 10- (C) weeks.
