# Supplementary material for: Formation of the junctions between lymph follicles in the Peyer's patches even before postweaning activation
Source: Sci Rep. 2024 Jul 9;14:15783. doi: 10.1038/s41598-024-65984-4 (PMC11233632; doi:10.1038/s41598-024-65984-4)
Supplement: Supplementary file 4 — Supplementary Video 3. [file 41598_2024_65984_MOESM4_ESM.pptx]

## Slide 1
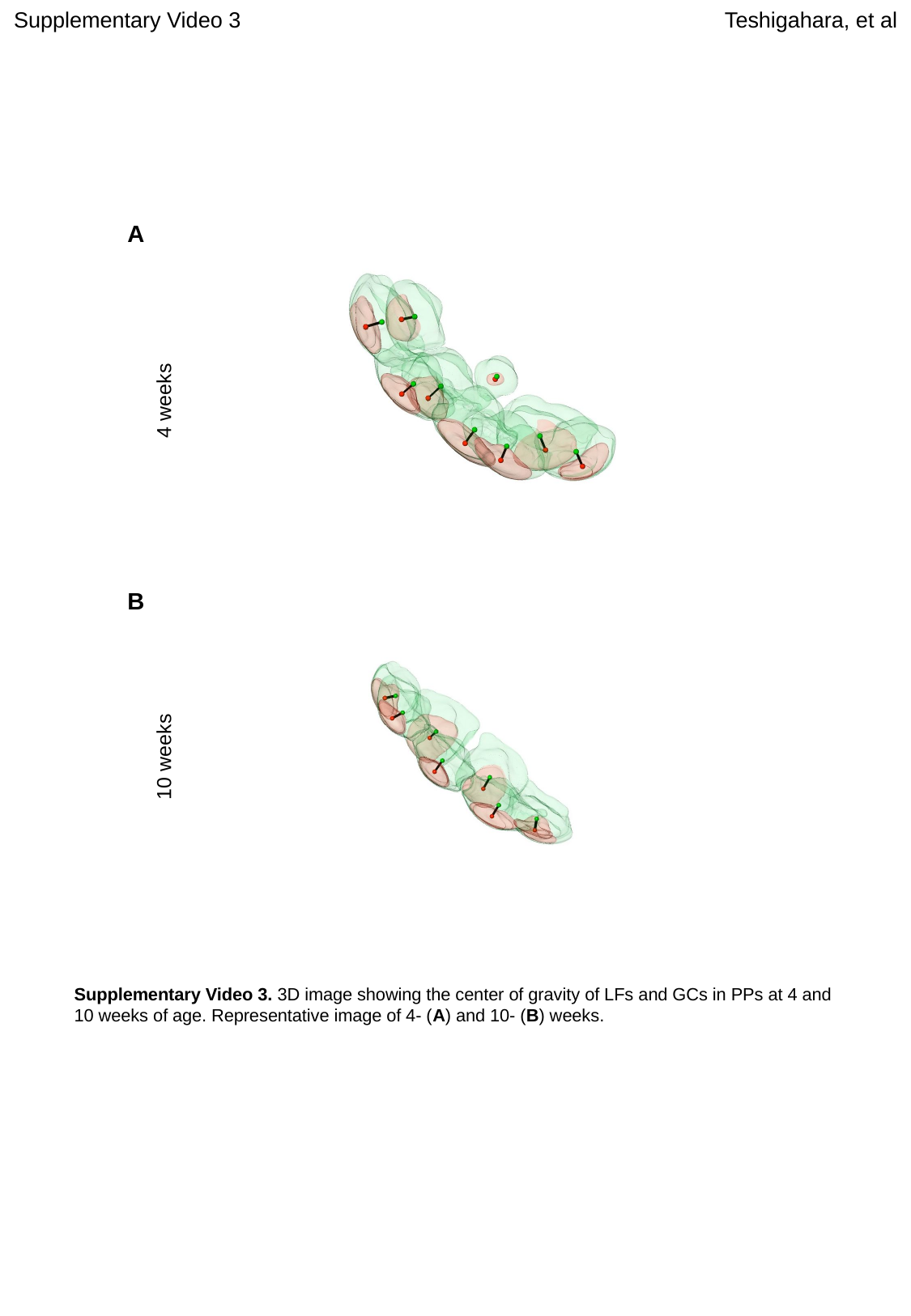

Supplementary Video 3
Teshigahara, et al
A
4 weeks
B
10 weeks
Supplementary Video 3. 3D image showing the center of gravity of LFs and GCs in PPs at 4 and 10 weeks of age. Representative image of 4- (A) and 10- (B) weeks.
